# Association between liver enzymes and type 2 diabetes: a real-world study

**DOI:** 10.3389/fendo.2024.1340604

**Published:** 2024-02-20

**Authors:** Yaru Bi, Yang Yang, Xiaojie Yuan, Jiping Wang, Tuo Wang, Zhiyuan Liu, Suyan Tian, Chenglin Sun

**Affiliations:** ^1^ Department of Endocrinology and Metabolism, First Hospital of Jilin University, Changchun, China; ^2^ Department of Health Examination Center, First Hospital of Jilin University, Changchun, China; ^3^ Department of Clinical Nutrition, First Hospital of Jilin University, Changchun, China; ^4^ Center for Applied Statistical Research, School of Mathematics, Jilin University, Changchun, China; ^5^ Department of Clinical Medicine, Yanbian University, Yanji, China; ^6^ Division of Clinical Research, First Hospital of Jilin University, Changchun, China

**Keywords:** alanine aminotransferase, aspartate aminotransferase, gamma-glutamyl transferase, type 2 diabetes, National Health and Nutrition Examination Survey, UK Biobank

## Abstract

**Aim:**

This study aimed to examine the association of liver enzymes, including alanine aminotransferase (ALT), aspartate aminotransferase (AST), and gamma-glutamyl-transferase (GGT), with type 2 diabetes (T2D) risk, particularly their dose-response relationship.

**Methods:**

This cross-sectional study enrolled participants aged >20 years old who underwent physical examination at our local hospital from November 2022 to May 2023. A generalized additive model (GAM) was fit to assess the dose-response relationship between liver enzymes and T2D risk. Furthermore, data from the UK Biobank (n=217,533) and National Health and Nutrition Examination Survey (NHANES 2011-2018; n= 15,528) were analyzed to evaluate whether the dose-response relationship between liver enzymes and T2D differed by population differences.

**Results:**

A total of 14,100 participants were included (1,155 individuals with T2D and 12,945 individuals without diabetes) in the analysis. GAM revealed a non-linear relationship between liver enzymes and T2D risk (*P*_non-linear_ < 0.001). Specifically, T2D risk increased with increasing ALT and GGT levels (range, <50 IU/L) and then plateaued when ALT and GGT levels were >50 IU/L. Elevated AST within a certain range (range, <35 IU/L) decreased the risk of T2D, whereas mildly elevated AST (>35 IU/L) became a risk factor for T2D. The UK Biobank and NHANES data analysis also showed a similar non-linear pattern between liver enzymes and T2D incidence.

**Conclusion:**

Liver enzymes were non–linearly associated with T2D risk in different populations, including China, the UK, and the US. Elevated ALT and GGT levels, within a certain range, could increase T2D risk. More attention should be given to liver enzyme levels for early lifestyle intervention and early T2D prevention. Further studies are necessary to explore the mechanism of the non-linear association between liver enzymes and T2D risk.

## Introduction

Diabetes is a common chronic metabolic disease characterized by sustained hyperglycemia with increasing prevalence worldwide. By 2045, 783 million adults worldwide are estimated to be living with diabetes (1), with type 2 diabetes (T2D) accounting for >90% (2). Long-term hyperglycemic status could increase the risk of developing life-threatening diabetic complications, such as cardiovascular and diabetic kidney diseases (3, 4). Therefore, identifying T2D risk factors is important for early intervention and delay of disease onset.

The liver plays an important role in controlling glucose homeostasis. The most common chronic liver disease is non-alcoholic fatty liver disease (NAFLD), which is characterized by excess fat accumulation in hepatocytes and is associated with T2D risk (5, 6), which might be reflected by increased liver enzymes. Circulating liver enzymes, such as alanine aminotransferase (ALT), gamma-glutamyl-transferase (GGT), and aspartate aminotransferase (AST), are non-invasive biomarkers of liver abnormalities. ALT is located predominantly in the cytoplasm and is the most specific marker of liver damage. Furthermore, ALT is associated with liver fat accumulation (7) and hepatic insulin resistance (8) and has been used as a surrogate marker for NAFLD in epidemiological studies (9, 10). GGT is present on the surface of most cell types and is widely used as an indicator of excessive alcohol intake and liver dysfunction. Moreover, recent studies have shown that GGT is related to components of metabolic syndrome, such as obesity (11), dyslipidemia (12), and hypertension (13). AST is a nonspecific liver enzyme involved in liver injury, and 80% of AST in the liver is present in hepatocyte mitochondria. Severe hepatocyte damage results in a significant increase in the serum AST levels (14).

Studies have investigated the association between liver enzymes and T2D risk; however, the results are controversial. Several studies revealed that ALT, AST, and GGT increase the risk of T2D (15, 16), whereas several studies showed that only ALT and GGT are risk factors for T2D (17–20). A study including 1441 individuals with a 7-year follow-up showed that AST alone is an independent risk factor for T2D after multivariable adjustment (21). Considering that China has the highest number of patients with T2D (1), only a few studies have investigated the association between liver enzymes and T2D risk in the Chinese population, particularly their potential dose-response relationship. Moreover, the distribution of liver enzymes may vary among different populations (22, 23), whether such differences translate to differences in the dose-response relationship between liver enzymes and T2D risk is still unknown.

In this study, we utilized large-scale physical examination data from China to investigate the association between liver enzymes (ALT, AST, and GGT) and T2D risk, particularly their dose-response relationship. Furthermore, the UK Biobank and National Health and Nutrition Examination Survey (NHANES) datasets were analyzed to determine whether the dose-response relationship differed by population differences and to validate the generalizability of the findings.

## Materials and methods

### Study participants

This was a cross-sectional study of 14,180 participants who underwent a routine physical examination at the Department of Health Examination Center of the First Hospital of Jilin University from November 2022 to May 2023. Participants aged >20 years were considered for inclusion in the study. Eighty individuals were excluded based on the following exclusion criteria: other types of diabetes, such as type 1 diabetes; excessive alcohol intake (>30 g/day for men and >20 g/day for women); liver injury with elevated liver enzymes 5-fold greater than the upper limit of normal (approximately ALT> 250 IU/L, AST>200 IU/L, GGT>300 IU/L); renal insufficiency with elevated serum creatinine 3-fold greater than the upper limit of normal (approximately >333 µmol/l); missing liver enzymes; missing fasting plasma glucose; self-reported liver cirrhosis and heart failure; pregnancy; cancer; and heart attack or acute infection in the last 3 months. A total of 14,100 participants (1,155 individuals with T2D and 12,945 individuals without diabetes) were included in the study.

### Data collection and measurements

Demographic and questionnaire data, including sex, age, smoking, alcoholic consumption, and medical history (hypertension and diabetes), were collected through an interview. Based on the smoking status, the participants were dichotomized as current and non-current smokers. Information on alcohol consumption was self-reported, and alcohol intake (g/day) was estimated based on the frequency and amount. The status of alcohol intake was divided into mild to moderate drinkers (<30 g/day for men and <20 g/day for women) and non-drinkers. Moreover, all subjects underwent anthropometric measurements, including height, weight, and blood pressure. body mass index (BMI) was calculated as weight (kg) divided by height squared (m^2^). After overnight fasting of 8–12 h, fasting plasma glucose (FPG), glycosylated hemoglobin (HbA1c), liver enzymes (ALT, AST, and GGT), serum lipid profile [total cholesterol (TC), triglyceride (TG), high-density lipoprotein cholesterol (HDL-C), and low-density lipoprotein cholesterol (LDL-C)] were assayed at the department of laboratory medicine. Furthermore, the presence of fatty liver was determined by abdominal ultrasound examination performed by professional experienced sonographers.

Hypertension was defined as a systolic blood pressure (SBP) ≥140 mmHg, diastolic blood pressure (DBP) ≥90mmHg or past medical history of hypertension. T2D was defined as self-reported T2D, FPG ≥7.0 mmol/l, or HbA1c ≥6.5%.

### Analysis of the UK Biobank and NHANES dataset

**Figure 1:**
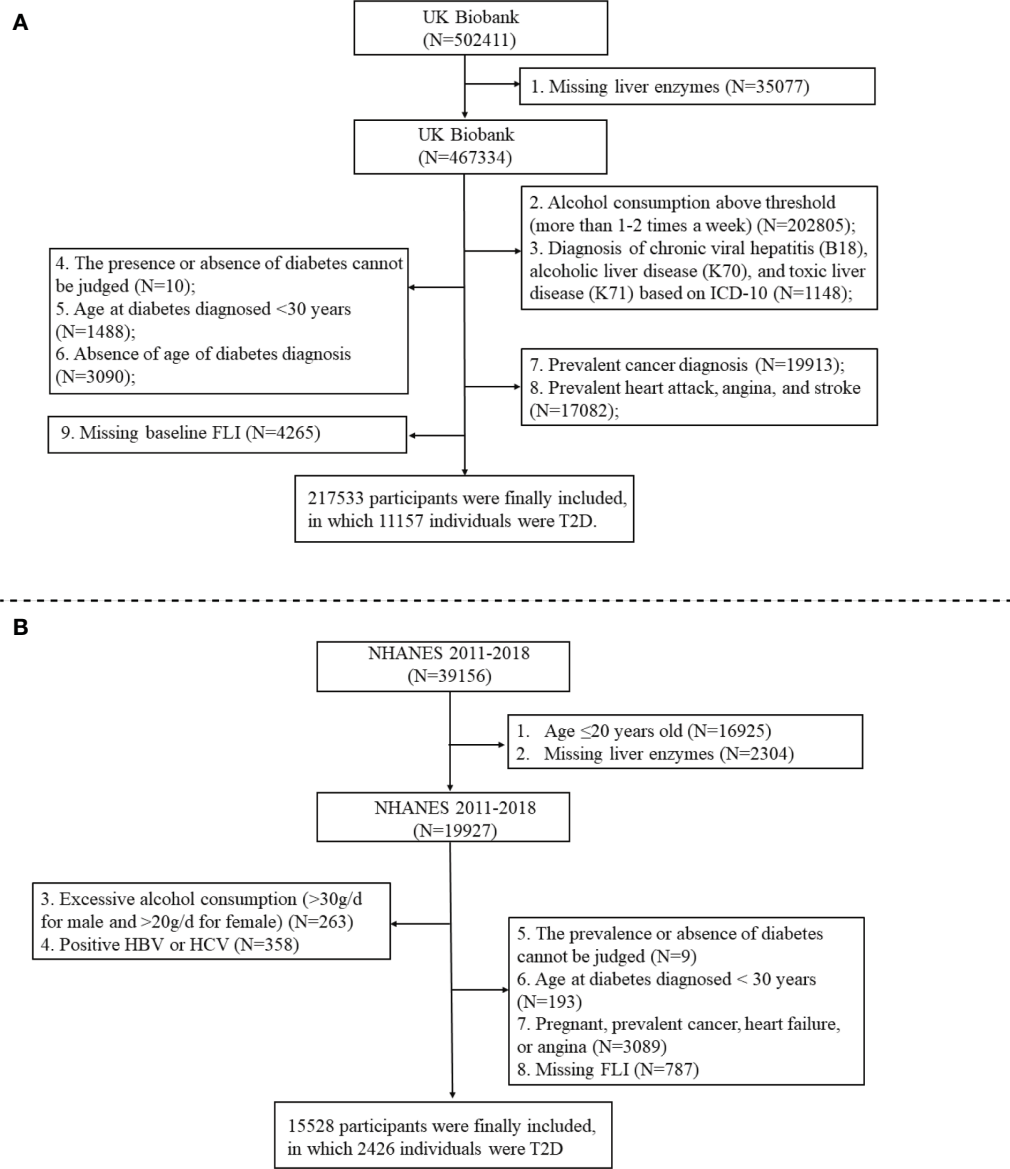
Flowchart of the participants selection in the UK Biobank **(A)** and NHANES **(B)**.

In addition to the Chinese dataset, we also analyzed the UK Biobank (application number 84347) from the UK population and the NHANES from the US population to reassess the dose-response relationship between liver enzymes and T2D risk. The UK Biobank is the world’s largest biomedical database. Between 2006 and 2010, >500,000 individuals aged 37-73 years were recruited into the cohort at baseline. Our study is a cross-sectional analysis of data from the UK Biobank, and the preliminary data extraction included 502,411 participants after application approval. The NHANES is a nationally representative cross-sectional survey of the noninstitutionalized US population to assess the health and nutritional status of children and adults. We conducted a cross-sectional analysis of data from NHANES 2011-2018 including 39,156 participants. **Figure 1** shows the flowcharts of the study population selection process for the UK Biobank and NHANES. We finally included 217,533 and 15,528 participants for UK Biobank and NHANES, respectively. **Supplementary File 1** provided details of the data collection, measurement, covariable, and T2D determination of the UN Biobank and NHANES.

### Statistical analysis

Continuous variables with normal distribution were expressed as mean ± standard deviation. Student’s t-test and analysis of variance were used for comparison between groups. Continuous variables with non-normal distribution were expressed as median (interquartile range). Mann–Whitney U–test or Kruskal–Wallis H-test was used for comparison between groups. Categorial variables were described as frequencies and percentages, and chi-square tests are used for the analysis of differences in distributions. The relationship between liver enzymes and T2D risk was examined using multivariable logistic regression after adjusting for potential confounding factors. Model 1 was adjusted for sex, age, BMI, hypertension, smoking, and drinking; model 2 was additionally adjusted for TC, TG, and the other two liver enzymes; model 3 was the same as model 2 with additional adjustment for NAFLD. Furthermore, generalized additive models (GAMs) were fit to evaluate the dose-response association between continuous liver enzyme levels and T2D risk in the Chinese dataset, UK Biobank, and NHANES dataset. *P* < 0.05 was considered statistically significant. Statistical analyses were performed using SPSS (version 22.0; IBM Corporation, Armonk, NY, USA), and GAMs were carried out in R software 4.1.0 (https://www.r-project.org/) using the R package “mgcv”.

## Results

We included 14,100 subjects (45.22% men) with a mean age of 45.43 ± 12.71 years who underwent conventional physical examination, including 1155 patients with T2D. All the data were complete without missing values except for HbA1c (3017 individuals with measured HbA1c). **Table 1** shows the baseline characteristics of the study population stratified by blood glucose. Compared with the non-diabetes control, the T2D group presented higher age, BMI, blood pressure, and worse serum lipid profile (higher TC, TG and LDL-C and lower HDL-C). The ALT, AST, and GGT levels in the T2D group were significantly higher than those in the non-diabetes group (*P* < 0.001).

**Table 1:** The characteristics of the study population stratified by blood glucose.

	All (N=14100)	Non-diabetes group (N=12945)	T2D group (N=1155)	*P*
Male, n (%)	6377 (45.22)	5634 (43.52)	743 (64.33)	<0.001
Age (years)	45.43±12.71	44.56±12.49	55.24±10.97	<0.001
BMI (kg/m^2^)	25.14±3.78	24.96±3.75	27.12±3.59	<0.001
WC (cm)	83.81±12.28	83.16±12.3	91.06±9.42	<0.001
SBP (mmHg)	130.51±17.75	129.35±17.13	143.45±19.39	<0.001
DBP (mmHg)	79.56±11.57	79.10±11.41	84.73±12.09	<0.001
Smoker, current	1980 (14.04)	1811 (13.99)	169 (14.63)	0.547
Drinking, mild to moderate	2848 (20.20)	2632 (20.33)	216 (18.70)	0.186
ALT (IU/L)	19.80 (14.00, 30.10)	19.30 (13.70, 29.30)	25.10 (18.20, 37.40)	<0.001
AST (IU/L)	21.70 (18.40, 26.60)	21.60 (18.40, 26.40)	23.0 (19.00, 30.00)	<0.001
GGT (IU/L)	24.00 (16.50, 39.20)	23.10 (16.10, 37.50)	36.10 (25.00, 56.00)	<0.001
FPG (mmol/L)	5.70 (5.40, 6.10)	5.13 (4.82, 5.49)	8.05 (7.24, 9.99)	<0.001
HbA1c (%) (N=3017)	5.29 (4.63, 6.00)	5.60 (5.30, 5.80)	7.20 (6.60, 8.00)	<0.001
TC (mmol/l)	5.35±1.09	5.22±1.02	5.52±1.22	<0.001
TG (mmol/l)	1.43 (1.00, 2.08)	1.24 (0.86, 1.83)	1.83 (1.29, 2.83)	<0.001
HDL-C (mmol/l)	1.34±0.31	1.38±0.31	1.27±0.28	<0.001
LDL-C (mmol/l)	3.35±0.77	3.25±0.75	3.46±0.83	<0.001
Fatty liver, n (%)	6791 (48.16)	5867 (45.32)	924 (80.00)	<0.001

BMI, body mass index; SBP, systolic blood pressure; DBP, diastolic blood pressure; ALT, alanine aminotransferase; AST, aspartate aminotransferase; GGT, gamma-glutamyl transferase; FPG, fasting plasma glucose; HbA1c, glycosylated hemoglobin; TC, total cholesterol; TG, triglyceride. HDL-C, high-density lipoprotein cholesterol; LDL-C, low-density lipoprotein cholesterol.

**Tables 2**–**4** list the baseline characteristics of the participants stratified by ALT, AST, and GGT quartiles. With the increase in quartiles of liver enzymes ALT, AST, and GGT, metabolic indicators, such as BMI, SBP, DBP, TC, TG, and LDL-C, increased gradually. Notably, FPG also increased progressively with the increase of quartiles of liver enzymes, which was statistically significant after inter-group comparison, indicating a positive association between liver enzymes and glucose metabolism.

**Table 2:** The characteristics of the study population stratified by ALT levels.

	Q1 (≤14) (N=3562)	Q2 (14.1-19.8) (N=3531)	Q3 (19.9-30.1) (N=3488)	Q4 (≥30.2) (N=3519)	P
Male, n (%)	569 (15.97)	1290 (36.53)	2003 (57.43)	2515 (71.47)	<0.001
Age (years)	42.10±13.10	48.06±12.83	47.86±12.18	43.76±11.62	<0.001
BMI (kg/m^2^) ^*^	22.83±3.05	24.59±3.29	25.78±3.34	27.40±3.85	<0.001
SBP (mmHg)^*^	124.32±16.75	130.63±18.28	133.08±17.88	134.10±16.38	<0.001
DBP (mmHg)^*^	75.72±10.43	78.84±11.52	80.93±11.48	82.84±11.61	<0.001
Hypertension, n (%) ^*^	654 (18.36)	1040 (29.45)	1221 (35.01)	1348 (38.31)	<0.001
AST (IU/L) ^*^	17.40 (15.60, 19.40)	20.30 (18.30, 22.50)	23.00 (20.60, 26.00)	30.80(26.30, 38.50)	<0.001
GGT (IU/L) ^*^	15.10 (12.50, 19.00)	20.8 (16.2, 27.8)	28.9 (21.2, 41.0)	45.7 (30.9, 71.1)	<0.001
FPG (mmol/l) ^*^	5.00 (4.72, 5.34)	5.17 (4.86, 5.60)	5.27 (4.92, 5.76)	5.34 (4.95, 5.91)	<0.001
HbA1c (%) (n=3017)	5.60 (5.30, 5.90)	5.70 (5.40, 6.00)	5.70 (5.50, 6.10)	5.80 (5.50, 6.40)	<0.001
TC (mmol/l) ^*^	4.98±0.95	5.24±1.01	5.32±1.07	5.43±1.08	<0.001
TG (mmol/l) ^*^	0.94 (0.71, 1.30)	1.17 (0.86, 1.67)	1.44 (1.01, 2.06)	1.76 (1.22, 2.56)	<0.001
HDL-C(mmol/l)	1.47±0.30	1.41±0.31	1.34±0.30	1.27±0.28	<0.001
LDL-C(mmol/l) ^*^	3.04±0.70	3.26±0.74	3.33±0.75	3.44±0.77	<0.001
Fatty liver, n (%)	694 (19.48)	1436 (40.67)	2003 (57.43)	2658 (75.53)	<0.001
T2D, n (%) ^*^	124 (3.48)	248 (7.02)	350 (10.03)	433 (12.30)	<0.001

*: Statistically significant pairwise comparison.

**Table 3:** The characteristics of the study population stratified by AST levels.

	Q1 (≤18.4) (N=3538)	Q2 (18.5-21.7) (N=3528)	Q3 (21.8-26.6) (N=3548)	Q4 (≥26.7) (N=3486)	P
Male (%)	935 (26.43)	1449 (41.07)	1826 (51.47)	2167 (62.16)	<0.001
Age (years)	38 (32, 51)	49 (37, 58)	49 (38, 57)	42 (34, 53)	<0.001
BMI (kg/m^2^) ^*^	22.83±3.05	24.59±3.29	25.78±3.34	27.40±3.85	<0.001
SBP (mmHg) ^*^	125.05±16.66	129.89±17.88	132.40±17.56	134.75±17.43	<0.001
DBP (mmHg)^*^	76.36±10.73	78.97±11.24	80.62±11.62	82.34±11.83	<0.001
Hypertension (%) ^*^	688 (19.45)	982 (27.83)	1212 (34.16)	1381 (39.62)	<0.001
ALT(IU/L) ^*^	12.30 (9.90, 15.50)	17.20 (13.90, 21.40)	26.20 (18.40, 39.98)	40.10 (29.48, 56.75)	<0.001
GGT(IU/L) ^*^	17.00 (13.30, 23.40)	21.40 (15.80, 30.70)	26.20 (18.40, 39.98)	41.10 (26.20, 66.70)	<0.001
FPG (mmol/l) ^*^	5.08 (4.77, 5.45)	5.15 (4.82, 5.58)	5.22 (4.89, 5.64)	5.32 (4.94, 5.85)	<0.001
HbA1c (%) (n=3017)	5.60 (5.30, 6.10)	5.60 (5.40, 6.00)	5.70 (5.40, 6.00)	5.70 (5.50, 6.30)	<0.001
TC (mmol/l) ^*^	4.96±0.94	5.21±1.03	5.33±1.04	5.46±1.09	<0.001
TG (mmol/l) ^*^	1.02 (0.75, 1.48)	1.18 (0.85, 1.69)	1.38 (0.96, 2.02)	1.63 (1.11, 2.45)	<0.001
HDL-C (mmol/l)	1.38±0.29	1.39±0.31	1.37±0.32	1.33±0.31	<0.001
LDL-C (mmol/l) ^*^	3.06±0.70	3.25±0.74	3.33±0.75	3.43±0.78	<0.001
Fatty liver, n (%)	1082 (30.58)	1508 (42.74)	1839 (51.83)	2362 (67.76)	<0.001
T2D, n (%)	238 (6.73)	250 (7.09)	275 (7.75)	392 (11.24)	<0.001

*: Statistically significant pairwise comparison.

**Table 4:** The characteristics of the study population stratified by GGT levels.

	Q1 (≤16.5) (N=3574)	Q2 (16.6-24.0) (N=3492)	Q3 (24.1-39.2) (N=3510)	Q4 (≥39.3) (N=3524)	P
Male, n (%)	382 (10.69)	1236 (35.40)	2067 (58.89)	2692 (76.39)	<0.001
Age (years)	39 (32, 51)	46 (35, 57)	48 (37, 57)	45 (36, 54)	<0.001
BMI (kg/m^2^) ^*^	22.60±2.90	24.54±3.28	26.12±3.41	27.34±3.73	<0.001
SBP (mmHg)^*^	123.05±15.82	129.69±17.73	133.68±17.72	135.73±16.98	<0.001
DBP (mmHg) ^*^	74.58±9.83	78.51±11.12	81.22±11.39	84.02±11.71	<0.001
Hypertension, n (%) ^*^	517 (14.47)	966 (27.66)	1287 (36.67)	1493 (42.37)	<0.001
ALT (IU/L) ^*^	12.60 (10.10, 16.20)	17.30 (13.60, 22.30)	23.00 (17.70, 31.10)	34.40 (24.50, 50.80)	<0.001
AST (IU/L) ^*^	18.80 (16.40, 21.70)	20.60 (17.90, 24.10)	22.60 (19.50, 26.90)	27.10(22.30, 34.50)	<0.001
FPG (mmol/l) ^*^	4.97 (4.70, 5.30)	5.14 (4.82, 5.51)	5.31 (4.96, 5.80)	5.38 (5.00, 6.03)	<0.001
HbA1c (%) (n=3017)	5.50 (5.30, 5.80)	5.60 (5.40, 6.00)	5.80 (5.50, 6.30)	5.80 (5.50, 6.48)	<0.001
TC (mmol/l) ^*^	4.95±0.95	5.17±1.02	5.30±1.03	5.55±1.08	<0.001
TG (mmol/l) ^*^	0.88 (0.67, 1.17)	1.15 (0.85, 1.58)	1.48 (1.06, 2.04)	1.91 (1.34, 2.82)	<0.001
HDL-C (mmol/l)	1.50±0.30	1.40±0.31	1.30±0.28	1.28±0.28	<0.001
LDL-C (mmol/l) ^*^	2.99±0.68	3.22±0.73	3.35±0.74	3.51±0.77	<0.001
Fatty liver, n (%)	546 (15.28)	1398 (40.03)	2183 (62.19)	2664 (75.60)	<0.001
T2D, n (%) ^*^	85 (2.38)	183 (5.24)	375 (10.68)	512 (14.53)	<0.001

*: Statistically significant pairwise comparison.

**Table 5** shows the odd ratios (OR) and corresponding 95% confidence intervals (CI) of T2D by quartiles of liver enzymes. In the crude model, elevated ALT, AST, and GGT levels increased T2D risk (top versus bottom quartile ALT: OR 3.89 [95% CI 3.17-4.78], AST: 1.76 [1.49-2.08], GGT 6.98 [5.52-8.82]). In model 1, with adjustment of age, sex, BMI, hypertension, smoking, and drinking, ALT and GGT were still risk factors of T2D, although the effect was attenuated. However, elevated AST levels were a protective factor of T2D. The association was evident after additionally adjusting for serum lipid (TC and TG), and the other two liver enzymes in model 2. With further additional adjustment for NAFLD in model 3, the results indicated that ALT and GGT levels in quartile 4 had an approximately 1.58-fold and 2.27-fold higher risk of developing T2D compared with ALT and GGT in the lowest quartile, respectively. The AST levels in quartile 4 could significantly decrease the risk of T2D (OR: 0.39; 95% CI:0.31-0.49).

**Table 5:** Multivariable logistic regression models assessing the relationship between liver enzymes and T2D.

ALT	Crude	Model 1	Model 2	Model 3
Q1 (≤14)	1	1	1	1
Q2 (14.1-19.8)	2.10 (1.68-2.61) [*P* < 0.001]	1.24 (0.98-1.57) [*P* = 0.074]	1.18 (0.94-1.50) [*P* = 0.161]	1.10 (0.87-1.40) [*P* = 0.434]
Q3 (19.9-30.1)	3.09 (2.51-3.82) [*P* < 0.001]	1.66 (1.32-2.08) [*P* < 0.001]	1.47 (1.17-1.86) [*P* = 0.001]	1.30 (1.02-1.64) [*P* = 0.033]
Q4 (≥30.2)	3.89 (3.17-4.78) [*P* < 0.001]	2.35 (1.87-2.97) [*P* < 0.001]	1.88 (1.44-2.46) [*P* < 0.001]	1.58 (1.21-2.08) [*P* = 0.001]
AST				
Q1 (≤18.4)	1	1	1	1
Q2 (18.5-21.7)	1.06 (0.88-1.27) [*P* = 0.052]	0.66 (0.54-0.80) [*P* < 0.001]	0.59 (0.48-0.72) [*P* < 0.001]	0.57 (0.46-0.69) [*P* < 0.001]
Q3 (21.8-26.6)	1.17 (0.97-1.40) [*P* = 0.097]	0.59 (0.48-0.71) [*P* < 0.001]	0.45 (0.37-0.56) [*P* < 0.001]	0.44 (0.36-0.54) [*P* < 0.001]
Q4 (≥26.7)	1.76 (1.49-2.08) [P<0.001]	0.83 (0.69-1.00) [*P* = 0.050]	0.41 (0.32-0.51) [*P* < 0.001]	0.39 (0.31-0.49) [*P* < 0.001]
GGT				
Q1 (≤16.5)	1	1	1	1
Q2 (16.6-24.0)	2.27 (1.75-2.95) [*P* < 0.001]	1.32 (1.00-1.73) [*P* = 0.052]	1.23 (0.93-1.61) [*P* = 0.150]	1.10 (0.84-1.46) [*P* = 0.486]
Q3 (24.1-39.2)	4.91 (3.86-6.24) [*P* < 0.001]	2.45 (1.89-3.17) [*P* < 0.001]	2.06 (1.59-2.68) [*P* < 0.001]	1.76 (1.35-2.30) [*P* < 0.001]
Q4 (≥39.3)	6.98 (5.52-8.82) [*P* < 0.001]	3.74 (2.88-4.86) [*P* < 0.001]	2.69 (2.04-3.54) [*P* < 0.001]	2.27 (1.71-3.01) [*P* < 0.001]

Model 1: adjusting for sex, age, BMI, hypertension, smoking, and drinking.

Model 2: adjusting for all factors in model 1 + TG +TC+ (AST+GGT for ALT analysis)/(ALT+GGT for AST analysis)/(ALT+AST for GGT analysis).

Model 3: adjusting for all factors in model 2 + NAFLD.

**Figure 2:**
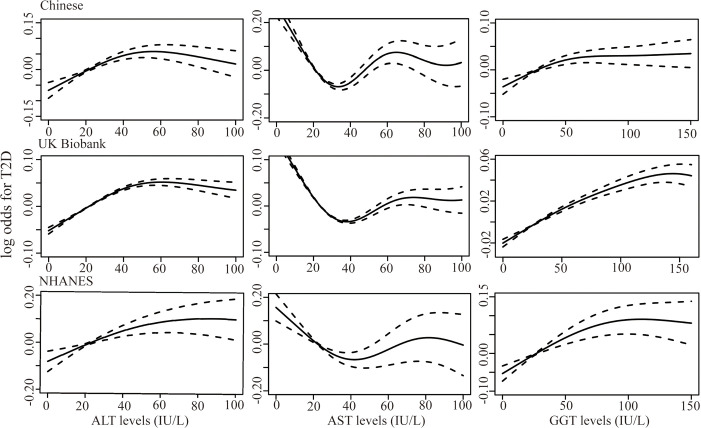
Generalized additive models were fit to assess the non-linear relationship between liver enzymes, ALT, AST, and GGT, and risk of T2D in the Chinese dataset (n=14100), UK Biobank (n=217533), and the NHANES dataset (n=15528) (all *P* < 0.001). X-axis represented the levels of liver enzymes and the y-axes represented the log odds for T2D.

**Figure 2** shows the results of the dose-response relationship between continuous liver enzyme levels and T2D risk. Overall, a non-linear association was identified between ALT, AST, and GGT and T2D risk (*P*_non-linear_ < 0.001) after adjustment for sex, age, BMI, hypertension, smoking, alcohol consumption, TC, TG, the other two liver enzymes, and NAFLD. Specifically, ALT and GGT <50 IU/L could increase the T2D risk, which plateaued when ALT and GGT levels were >50 IU/L. Elevated AST levels <35 IU/L played a protective role in T2D development, whereas mildly elevated AST at >35 IU/L could also be a risk factor of T2D.

The dose-response relationship between liver enzymes and T2D risk in the UK and US populations was also analyzed. We included 217,533 and 15,528 participants from the UK Biobank and NHANES in the analysis, including 11,157 (5.13%) and 2426 (15.62%) patients with T2D, respectively. We identified a similar non-linear pattern between liver enzymes and T2D risk in UK and US populations (*P*_non-linear_ < 0.001, **Figure 2**). The ALT and AST levels, corresponding to the peak and bottom risk of T2D, were 50 and 35 IU/L, respectively, which was consistent with the results from the Chinese dataset. However, in the UK Biobank, elevated GGT levels within 125 IU/L could increase the T2D risk, then plateaus thereafter, which was higher than that in the Chinese and NHANES datasets. In the GAMs, potential confounding factors, including sex, age, BMI, hypertension, physical activity (International Physical Activity Questionnaire for UK Biobank and categorical physical activity, including vigorous, moderate physical activity and inactivity for NHANES), family history of diabetes, smoking, drinking, TG, TC, other two liver enzymes, and NAFLD were adjusted.

## Discussion

We investigated the association of liver enzymes with T2D risk. We identified a non-linear relationship of ALT, AST, and GGT with T2D after controlling for potential confounding factors. T2D risk was continuously increasing with increasing ALT and GGT levels within a certain range. However, T2D risk plateaued when ALT and GGT increased to a certain degree. Elevated AST levels within a certain range initially exhibited a protective role, and mildly elevated AST levels were a risk factor of T2D. Additionally, the UK Biobank and NHANES dataset verified the non-linear association between liver enzymes and T2D risk, indicating a credible result for the Chinese dataset. Moreover, the non-linear association was scarcely affected by population differences.

Our findings were generally consistent with previous studies in South Korea (18, 19), Japan (24), Iran (25), and Germany (26), and we expanded previous reports by assessing the dose-response association of ALT and GGT with T2D risk. Elevated ALT and GGT levels within 50 IU/L could increase T2D risk, and T2D risk plateaued (“saturation effect”) when ALT and GGT levels were > 50 IU/L for Chinese populations. Although the genetic background and distribution of liver enzymes were different in different populations, a similar non-linear pattern was identified in the UK and US populations. However, the cutoff value of GGT in the UK Biobank was higher than that in the Chinese and NHANES dataset. Our findings were consistent with one meta-analysis conducted only on GGT and T2D risk, which also showed a non-linear association (27).

The findings have clinical significance as follows. First, the cutoff values of ALT and GGT at 50 IU/L could be considered the upper limit of the normal reference range in the general population, suggesting that increased ALT and GGT levels within their normal ranges are risk factors of T2D. One individual had elevated ALT and GGT levels (<50 IU/L) as compared to previous ones, indicating increased risk of T2D even though the glucose metabolism indicators such as FPG were within normal range. Therefore, early lifestyle intervention is needed for early T2D prevention. Second, measurements of liver enzymes are sensitive, standardized, and economical in clinical practice. ALT and GGT may clinically serve as useful markers in identifying individuals with higher T2D risk.

The association between AST and T2D risk is uncertain (17, 21, 25, 28, 29). Our study used continuous AST values and identified a non-linear association between AST and T2D risk, which was also verified in two other large-scale datasets. One study, including 9895 subjects, showed that elevated AST levels were a risk factor of diabetes (elevated versus normal AST; OR:1.75 [1.32-2.32]), whereas AST within the normal ranges of quartiles 2, 3, and 4 was negatively associated with T2D risk compared with quartile 1 (Q2, Q3, Q4 versus Q1, OR<1, 95% CI <1) (25), indicating that the association between AST and diabetes risk may be a non-linear trend of initially decreasing followed by an increasing trend. The non-linear association between continuous AST levels and T2D risk was revealed for the first time, and more studies are necessary to help provide the mechanisms for the non-linear association.

Although the mechanism underlying the positive association of ALT and GGT with T2D risk is unclear, it could be explained by ALT and GGT both reflecting fat accumulation in the liver (7, 30), resulting in hepatic insulin resistance, causing increased hepatic glucose production. These pathophysiological changes are involved in the pathogenic mechanism of T2D (31). In our study, we adjusted fatty liver in the GAMs, and the results still showed a positive association of ALT and GGT with T2D. Additionally, a study including 3556 individuals without ultrasound-diagnosed fatty liver also indicated that increased ALT and GGT levels are risk factors for T2D (32). This suggests that ALT and GGT are independent risk factors of T2D independent of fatty liver, and extra mechanisms might exist accounting for the positive association. GGT plays an essential role in maintaining the production of intracellular antioxidant glutathione, also considered a biomarker of oxidative stress (33). Furthermore, elevated ALT and GGT levels are associated with chronic inflammation (34, 35). Oxidative stress, together with inflammation, could damage the insulin signal pathway, which contributes to the development of T2D (36–38).

Furthermore, we hypothesize the following conjecture to account for the non-linear association between ALT and GGT and T2D risk. The regulation of blood glucose homeostasis is involved in multiple organs, especially the liver (39). It may cause minor liver damage when the liver tries to maintain glucose homeostasis, which clinically manifests as elevated ALT and GGT levels, even within their roughly normal ranges. Thus, elevated ALT and GGT levels within a certain range may be the consequences of self-regulation by the liver to maintain glucose homeostasis. However, when abnormal glucose homeostasis develops to a certain extent wherein the liver is unable to maintain glucose homeostasis at the cost of damaging itself (elevated ALT and GGT within a certain range), the association of ALT and GGT with T2D risk disappears. Further investigation, however, is required to test this research hypothesis.

The strengths of the present study are twofold. First, extensive large-scale data from up to 14,100 individuals were collected, and the dose-response relationship between liver enzymes and T2D in the Chinese population was systematically explored, which was rarely studied and poorly understood. Second, the analysis and comparison of three large-scale datasets comprehensively offered a significant advantage. Thus, it increased the credibility and generalizability of the findings, and the strategy could detect the possible population differences of the findings.

This study has several disadvantages. First, the participants in the Chinese dataset did not undergo virologic testing; therefore, we cannot exclude individuals with positive viral hepatitis, which may be a confounder of this association. Second, the history of alcohol consumption was self-reported, and information bias may be present. Third, we could not fully adjust the potential confounding factors or exclude residual confounding factors. For example, although ultrasound has been suggested as a first-line screening tool for defining liver steatosis in the general population (40), it is less sensitive when liver fat is <30% (41); therefore, a small part of an individual with mild fatty liver may be neglected. Fourth, we could not comprehensively explore the association of liver enzymes with the onset and development of T2D, as the course of self-reported T2D was not recorded. Finally, the participants were from the northeast of China, which could not represent the characteristics of the overall Chinese population.

In conclusion, liver enzymes ALT, AST, and GGT presented a non-linear pattern with T2D risk after adjustment for comprehensive diabetes risk factors, which was generalizable to different populations from China, the UK, and the US. Higher ALT and GGT levels, within a certain range, were indicative of the development of T2D. More attention should be given to liver enzymes levels for early lifestyle intervention and early T2D prevention. Meanwhile, more studies are warranted to explore the underlying mechanisms for the non-linear association between liver enzymes and T2D risk.

## Data availability statement

The original contributions presented in the study are included in the article/**Supplementary Material**. Further inquiries can be directed to the corresponding authors.

## Ethics statement

The studies involving humans were approved by Ethics Committee of the First Hospital of Jilin University. The studies were conducted in accordance with the local legislation and institutional requirements. The participants provided their written informed consent to participate in this study.

## Author contributions

YB: Data curation, Investigation, Methodology, Writing – original draft. YY: Data curation, Formal analysis, Writing – original draft. XY: Data curation, Methodology, Resources, Writing – original draft. JW: Data curation, Methodology, Software, Writing – original draft. TW: Data curation, Methodology, Software, Writing – original draft. ZL: Methodology, Writing – original draft. ST: Conceptualization, Visualization, Writing – review & editing. CS: Conceptualization, Visualization, Writing – review & editing.
