# Supplementary material for: Association between liver enzymes and type 2 diabetes: a real-world study
Source: Front Endocrinol (Lausanne). 2024 Feb 20;15:1340604. doi: 10.3389/fendo.2024.1340604 (PMC10913017; doi:10.3389/fendo.2024.1340604)
Supplement: Supplementary file 1 [file DataSheet_1.docx]

**The data collection and measurement, exposure, outcome, and covariables in the UK Biobank and NHANES datasets**

**UK Biobank**

**Date collection and measurements**

In the UK Biobank, all participants were interviewed using a touch-screen to collect the information about sociodemographic characteristics, lifestyle, medical history, among others. Moreover, physical measures such as blood pressure and anthropometry are measured by qualified research nurses. Blood samples were also collected from the participants who agreed to provide the samples for analysis. We extracted data including sex, age, ethnicity, BMI, blood pressure, waist circumstance (WC), smoking, alcohol consumption, medical history (such as hypertension, cancer, heart attack, angina, and stroke), physical activity, glucose, HbA1c, liver enzymes (ALT, AST and GGT), and serum lipid profile (TC, TG, HDL-C, and LDL-C). The UK Biobank was approved by the North West Multi-Centre Research Ethics Committee and all participants provided the informed consent.

**Exposure and the outcome variable**

Liver enzymes are measured using enzymatic rate method (Beckman Coulter AU5800, Beckman Coulter, Ltd). Diabetes was ascertained based on the questionnaire “Has a doctor ever told you that you have diabetes”, For undiagnosed diabetes, the individuals with the measurement of HbA1c ≥48 mmol/mol or random glucose ≥11.1 mmol/l were also be considered as diabetes. To minimize the number of individuals with type 1 diabetes, rather than T2D, we excluded diabetic individuals who reported that they are diagnosed under the age of 30 years and those who are absence of age of diabetes diagnosis.

**Covariates**

Race was grouped as white ethnicity (accounting for 94.14%) and the others. The participants with alcohol consumption above the threshold (more than 1-2 times per week) were regarded as excessive alcohol consumption and were excluded for analysis. For all other participants, the status of alcohol intake was categorized as 1-2 times/week, 1-3 times/month, and occasions/never. Smoking status was divided into current, former and never. Physical activity was evaluated according to International Physical Activity Questionnaire (IPAQ) and can be grouped into low, moderate, and high. Hypertension was defined based on the 2-time average measured blood pressure (systolic blood pressure ≥140mmHg or diastolic blood pressure ≥90mmHg) or past medical history of hypertension diagnosed by doctors. No imaging, ultrasonography data regarding fatty liver in the UK Biobank. Therefore, NAFLD is defined using the fatty liver index (FLI), which was calculated through four variables including BMI, WC, GGT, and TG listed as follows. FLI was categorized according to Bedogin et al. as follows, a FLI <30 rules out fatty liver, a FLI between 30 to 59 rules in intermediate status, and FLI ≥60 rules in fatty liver.

$$\mathrm{FLI}=100\times\frac{e^{0.953\times\ln\left( TG \right)+0.139\times BMI+0.718\times\ln\left( GGT \right)+0.053\times Waist-15.745}}{1+e^{0.953\times\ln\left( TG \right)+0.139\times BMI+0.718\times\ln\left( GGT \right)+0.053\times Waist-15.745}}$$

**NHANES**

**Date collection and measurements**

In the NHANES, participants underwent in-home interviews, health examinations, and laboratory tests at mobile examination centers. We extracted demographics data (sex, age, race), examination data (blood pressure, BMI, waist circumference), laboratory data (ALT, AST, GGT, TC, TG, HDL-C, LDL-C, blood glucose, HbA1c, liver disorder-related indicators), and questionnaire data (alcohol use, smoking status, medical history of hypertension and diabetes, physical activity) from the NHANES dataset. NHANES was approved by the National Center for Health Statistics Research Ethics Review Board. All the participants provided the informed consents.

**Exposures and the outcome variables**

Liver enzymes are measured using standard procedure. Diabetes was ascertained by FPG (≥7.0 mmol/l), OGTT-2h glucose (≥11.1 mmol/l), HbA1c (≥6.5%), or the use of hypoglycemia medication or insulin. As similar to the ascertainment of T2D in the UK Biobank, we excluded diabetic individuals who reported that they are diagnosed under the age of 30 years to minimize the number of individuals with type 1 diabetes.

**Covariate variable**

Race was categorized as Mexican American, other Hispanic, Non-Hispanic, or the other race. Alcohol intake was quantified based on the amount and the frequency of alcoholic consumption, and the participants with excess alcohol intake (>30g/d for male and >20g/d for female) were excluded. The other participants were categorized as current drinker and non-drinker. The status of smoking was classified as every day, some days, and never. Physical activity was dichotomized vigorous, moderate physical activity and inactivate. Hypertension was defined based on the 2-time average measured pressure (systolic blood pressure ≥140mmHg or diastolic blood pressure ≥90mmHg) or taking prescription for hypertension. Fatty liver was determined by FLI as described in the UK Biobank. A positive hepatitis B surface antigen and a positive hepatitis C RNA are deemed as being positive HBV and HCV, which are excluded from the final analysis.
